# Variation in digital breast tomosynthesis image quality at differing heights above the detector

**DOI:** 10.1002/jmrs.565

**Published:** 2021-12-27

**Authors:** Rob Davidson, Khaled Al Khalifah, Abel Zhou

**Affiliations:** ^1^ Discipline of Medical Radiation Science University of Canberra Bruce Australian Capital Territory 2615 Australia; ^2^ Radiologic Sciences Department Kuwait University Sulaibekhat Kuwait

**Keywords:** Breast phantom, breast tomosynthesis, contrast‐to‐noise ratio, figure‐of‐merit, image quality

## Abstract

**Introduction:**

The aim of this preliminary work was to determine if image quality in digital breast tomosynthesis (DBT) changes when tomosynthesis image slices were obtained at differing heights above the detector and in differing breast thicknesses.

**Methods:**

A CIRS Model 020 BR3D breast imaging phantom was used to obtain the DBT images. The images were also acquired at different tube voltages, and each exposure was determined by the automatic exposure control system. Contrast‐to‐noise ratio (CNR) and figure‐of‐merit (FOM) values were obtained and compared.

**Results:**

At a phantom thickness of 5 cm or greater, there was a significant reduction (*P* ≤ 0.05) of image CNR values obtained from the images near the top of the phantom to those obtained near the bottom of the phantom. When the phantom thickness was 4 cm, there was no significant difference in CNR values between DBT images acquired at any height in the phantom. FOM values generally showed no difference when images were obtained at differing heights above the detector.

**Conclusion:**

Image quality, as measured by the CNR, was reduced when tomosynthesis slice image heights were at the top of the phantom and when the thickness of the phantom was more than 4 cm. From this preliminary work, clinicians need to be aware that DBT images obtained near the top of the breast, when breast thickness is greater than 4 cm, may have reduced image quality. Further work is needed to fully assess any DBT image quality changes when images are obtained near the top of the breast.

## Graphical abstract

Digital breast tomosynthesis (DBT) image quality has been assessed at different slice heights above the detector within a phantom. This initial work has shown that image quality differs when DBT slice image heights were at the top of the phantom compared to the bottom and when the phantom was greater than 4 cm thick. Clinicians need to be aware of this preliminary finding as a potential source of false negative findings when using DBT.

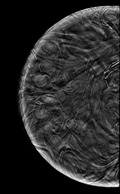

## Introduction

In 2011, the U.S. Food and Drug Administration approved the use of digital breast tomosynthesis (DBT) which is rapidly increasing worldwide. DBT has been found to be superior to digital mammography (DM) in diagnostic settings for early detection and has improved diagnostic ability of breast cancer detection^1^, ^2^, ^3^, ^4^, ^5^ and improved outcomes from breast screening programs.^6^, ^7^, ^8^, ^9^ It has been approved for use as an adjunct to two‐dimensional (2D) mammography for breast cancer screening in several countries^8^, ^9^ and has been investigated in several large prospective population‐based trials.^10^

Digital breast tomosynthesis produces a stack of 2D image slices and a pseudo three‐dimensional (3D) image set, of the breast by taking multiple low‐dose images per view or projections along an arc over the breast.^11^, ^12^ The Xray tube moves continuously or in a step‐and‐shoot motion in a 15–50° arc obtaining 9–25 projections.^1^, ^13^ These projected planar images are used to create tomosynthesis images that are parallel to the detector, and which vary in image thickness/slice sensitivity profile (SSP) depending on the arc angle and the number of projections.^14^ The tomosynthesis images reduce the superimposition of different breast structures, offering a superior detection accuracy in breast cancer screening.^15^

Image quality of the DBT images, similar to 2D mammography, is determined by many factors, including the selected X‐ray tube target materials, such as molybdenum (Mo), rhodium (Rh), or tungsten(W), and the X‐ray tube filtration materials, such as silver (Ag), aluminium (Al), Mo, or Rh; X‐ray beam quality. Furthermore, image quality is also determined by the detector types, such as amorphous selenium [a‐Se], caesium iodine (CsI) and the detector pixel size. In addition, DBT image quality is also affected by pixel binning, angular range, the number of projection images, X‐ray tube motion, detector motion, image reconstruction algorithms, image post‐processing and the total amount radiation delivered or dose received in the examination.^16^ In DBT, since the image contrast can be manipulated, the contrast‐to‐noise ratio is a main image quality index.^17^, ^18^, ^19^, ^20^, ^21^

Research is ongoing into image quality and improvements in DBT. Factors that affect object blur and visibility are focal spot size^22^, ^23^ and scatter radiation.^24^, ^25^ Breast thickness and density affect the scatter‐to‐primary ratio (SPR), and increasing SPR reduces image quality.^26^ This study by Rodrigues et al.^26^ also showed that the best overall image quality was obtained with 30° of the angular range and 15 projection images combined with a W–Ag target filter. The focus of these studies has been on overall image quality, not at specific heights above the receptor. However, a study by Jousi et al^16^ showed that as the DBT angle increased, objects become blurred, and the visibility of small objects, such as microcalcifications, decreased. Cockmartin et al^27^ found that microcalcification detection became worse in DBT than DM when breast thicknesses was 40 mm and greater and when the DBT image slice was 20 mm and greater, above the height of the detector. Shaheen et al^28^ showed in their simulation experiment that contrast of spheres and the signal difference‐to‐noise ratio (SDNR) reduced at increasing heights above the detector. Furthermore, anecdotal evidence from clinical mammographic radiographers also suggested that when the DBT image slice was further from the detector, especially in larger breast sizes, there is greater blurring of microcalcifications and potential to not visualise small pathologies.^29^ No other discussion could be found in the literature relating to the change in image quailty at various tomosynthesis image slice heights above the detector in a DBT 3D data set, and as such changes are related to breast thickness.

The aim of this preliminary research was to determine if image quality in DBT changed when tomosynthesis image slices were obtained at differing heights above the detector and in differing breast thicknesses.

## Method

### Materials

The unit that was used in this study was the Hologic Selenia Dimensions (Hologic Inc., Marlborough, USA) digital mammographic (DM) unit installed in BreastScreen ACT, Canberra, Australia. The mammography unit has a tungsten (W) target and selectable filter materials of rhodium (Rh) and silver (Ag) available for 2D imaging. The unit is equipped with an amorphous selenium (a‐Se) detector being 24 × 30 cm (10 × 12 inch) in size with 70 µm pixel size. The unit is fitted with tomosynthesis which uses an aluminium (Al) filter and no grid during DBT image acquisition. The tomosynthesis images are acquired using a continuous motion arc swing over a 15° with 15 projections acquired in 3.7 seconds exposure time, where the X‐rays are pulsed on and off during the acquisition time. The resultant tomosynthesis images have a pixel size of approximately 100 µm and 1 mm slice thickness. This height or distance of the compression paddle to the image receptor is a measure of the compressed breast thickness. The number of slices reconstructed for each mammographic examination depends on the compression paddle height above the image receptor.

The mammographic unit selected for this study meets the standards of BreastScreen Australia digital mammography imaging system performance^30^ and the Royal Australian and New Zealand College of Radiologists (RANZCR) accreditation^31^ for clinical mammography examinations.

The phantom selected for use in this study was the CIRS Model 020 BR3D Breast Imaging Phantom (CIRS Inc., Norfolk, USA). This phantom was selected as other works, not yet reported, will use human observers to evaluate image quality of the same images. The CIRS phantom is one of a few specifically designed phantoms for DBT^32^ with materials designed to mimic adipose and gland tissues ‘swirled’ together. The CIRS phantom has previously been shown to be a valid phantom for this type of research where it was also been used where signal measurements have been taken.^33^ Furthermore, a similar phantom to the CIRS phantom with ‘cloud‐like’ appearance rather than ‘swirls’, also manufactured by the CIRS Inc, has been used to measure the contrast‐to‐noise (CNR) ratio in DBT images.^34^

**Figure 1:**
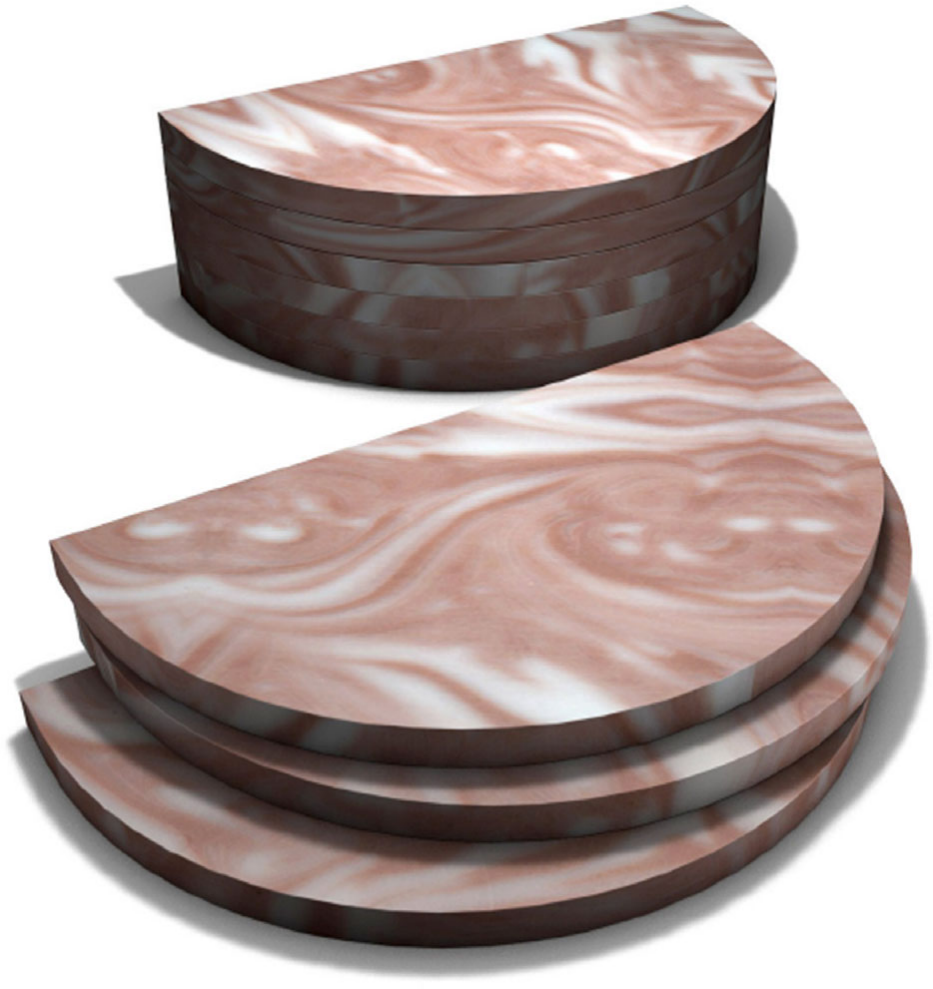
CIRS Model 020 BR3D Breast Imaging Phantom showing two tissue‐equivalent materials ‘swirled’ together that mimic adipose and glandular tissues. Three individual slabs are shown and one configuration of the phantom using all six slabs.

An image of the phantom and individual slabs can be seen in Figure 1. The CIRS phantom consists of six semi‐circular shaped 100 × 180 × 10 mm slabs made of epoxy resin. Each slab contains two tissue‐equivalent materials that mimic adipose and glandular tissues that are ‘swirled’ together to mimic heterogeneous breast tissue. Five of the slabs are background slabs, and one slab is the target slab with calcium carbonate (CaCO_3_) specks and fibres and spheroidal masses to mimic breast carcinoma. Figure 1 also shows one of the phantom’s configurations with six slabs together to form a 6 cm thick phantom.

**Figure 2:**
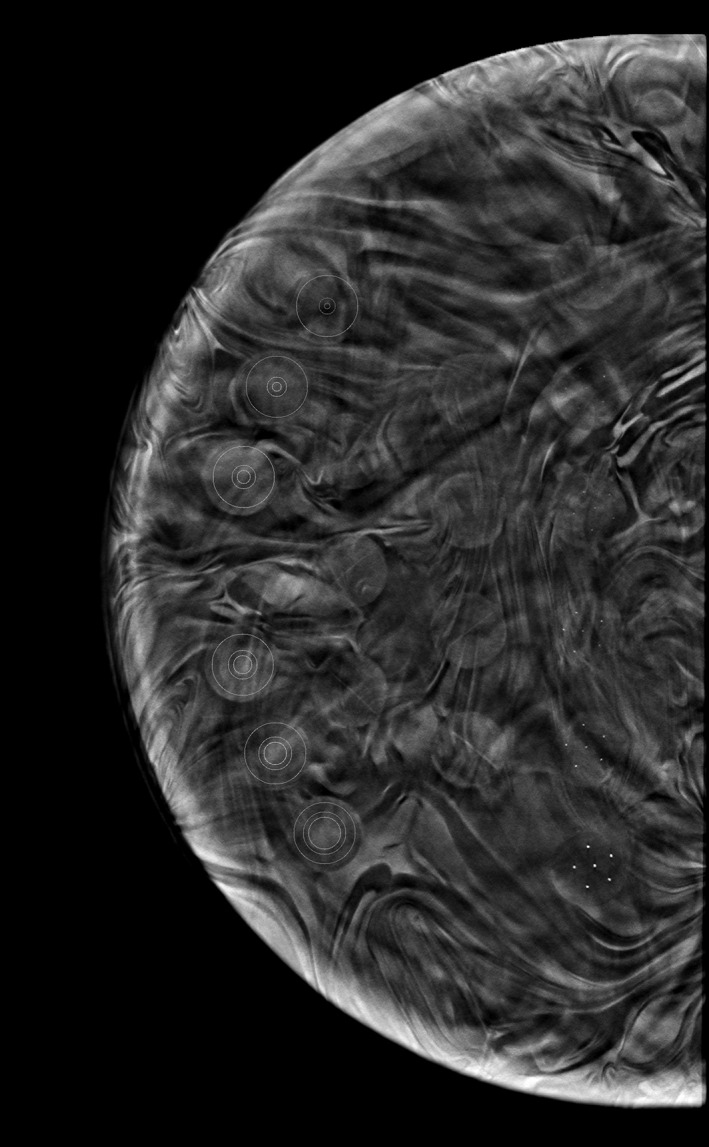
Digital breast tomosynthesis (DBT) image slice of the target slab of the Breast Imaging Phantom. Circle and annulus regions of interest for object and background measurements, respectively, are located over the spheroidal masses.

In the target slab, there are six spheroidal masses with sizes ranging from 6.3 mm to 1.8 mm in diameter. These masses were selected to measure the CNR and to calculate the figure‐of‐merit (FOM). Figure 2 shows a tomosynthesis image slice of the CIRS phantom target slab. The circle and annulus regions of interest (ROI) placed over each of the spheroidal masses, shown on the phantom image in Figure 2, were used for measurement of the signal and background pixel values, respectively.

### Image acquisition

Digital breast tomosynthesis images of the CIRS breast imaging phantom were obtained. The phantom was placed on the DM unit with thicknesses of 4, 5 and 6 cm. Table 1 shows the technical factors that were used to obtain the tomosynthesis images of the CIRS breast imaging phantom at the three thicknesses of the phantom. At each phantom thickness, the X‐ray tube voltage (kVp) settings that were used were based on clinical advice of the typical kVp settings that could be used for the equivalent breast thicknesses. At each phantom thickness, the target slab was located at the bottom, middle (which varied in location due to the phantom thickness) and at the top of the phantom. For each image acquisition, that is, at each phantom thickness and kVp setting, the tube current–exposure time product (mAs) was determined by the automatic exposure control system. Entrance skin dose (ESD) in milli‐Gray (mGy), organs/absorbed dose in milli‐Gray (mGy) and exposure index (EI) values were recorded. All images were transferred directly to the researchers’ PC in the DICOM format for analysis.

**Table 1:** Tube voltage settings and location of the target slab used at the three CIRS breast imaging phantom thicknesses.

CIRS phantom thickness	Tube voltage setting (kVp)	Target slab location (cm above image receptor)
4 cm	28, 30, 32	1, 2, 4
5 cm	28, 30, 32, 34	1, 3, 5
6 cm	30, 32, 34	1, 4, 6

### Analysis

Ten tomosynthesis images, approximately 1 mm thick, were obtained from each 10 mm thick target phantom slab, under each phantom thickness and exposure condition. Three adjacent images from the target slab, obtained at each setting and location, were selected by three^3^ radiographers, with a mean clinical experience of approximately 27 years, representing the optimal tomosynthesis quality of the target slab objects. These three images, at each setting and target slab location, were used in the image analysis for measurements of the CNR and FOM.

Image analysis was undertaken by measuring pixel values in the six contrast discs and background of the image. Puett et al used a similar methodology using a CIRS Model 020 BR3D phantom.^33^ Images were imported into MatLab Ver. R2018b (The MathWorks Inc., Natick, Massachusetts) and opened for viewing. The ROI were defined based the size of the spheroidal masses of the object and background within the image (see Fig. 2). The ROI area of the masses, used to measure the object pixel values, ranged from 0.9 mm^2^ (approx. 10 pixels) to approximately 23.4 mm^2^ (approx. 266 pixels). Around each mass, a background anulus ROI was defined. The anulus area used to measure the background pixel values ranged from approximately 74.3 mm^2^ (approx. 844 pixels) to 33.5 mm^2^ (approx. 380 pixels). The size of the ROI for the object and background was constant for all images. The location of the ROI for the object and background was manually adjusted for each tomosynthesis image.

Mean and standard deviation of the pixel values in each object and background ROI were recorded and used to obtain the CNR.^34^, ^35^, ^36^, ^37^ The CNR is one measure of image quality and was calculated using.

(1) $$CNR=\frac{\left|M_{O}‐M_{\mathrm{BG}}\right|}{\sigma_{\mathrm{BG}}},$$

where, M_O_ is the mean pixel value of the object, M_BG_ is the mean pixel value of the background *and* σ_BG_ is the standard deviation of the background.

The FOM is a measure of image quality that is independent of the amount of radiation or dose used to acquire the image, defined as^20^, ^37^, ^38^ and was calculated using.

(2) $$FOM=\frac{{\mathrm{CNR}}^{2}}{\mathrm{AGD}},$$

where, CNR is measured using equation (1) and AGD is the average glandular dose (mGy) recorded by the mammography unit

Measurements of the CNR and calculation of FOM values were obtained from each of the six spheroidal masses, and the mean and standard error of the means (SEM) were calculated for each phantom thickness and each tomosynthesis image slice height above the detector. Statistical analysis was undertaken using one‐way analysis of variance (ANOVA) with Tukey’s post hoc method to compare if the mean CNR and FOM values differed when the DBT images of the target slab, using different tube voltage settings, were obtained at different heights above the detector and at different phantom thicknesses. Significance was set at *P* ≤ 0.05.

## Results

A total of 30 DBT image acquisitions were obtained at various tube voltage settings and CIRS phantom thicknesses. At each tube voltage setting and phantom thickness, there was no significant difference (*P* > 0.05) between the tube current‐exposure time product, AGD, ESE and EI values of each image acquisition.

**Figure 3:**
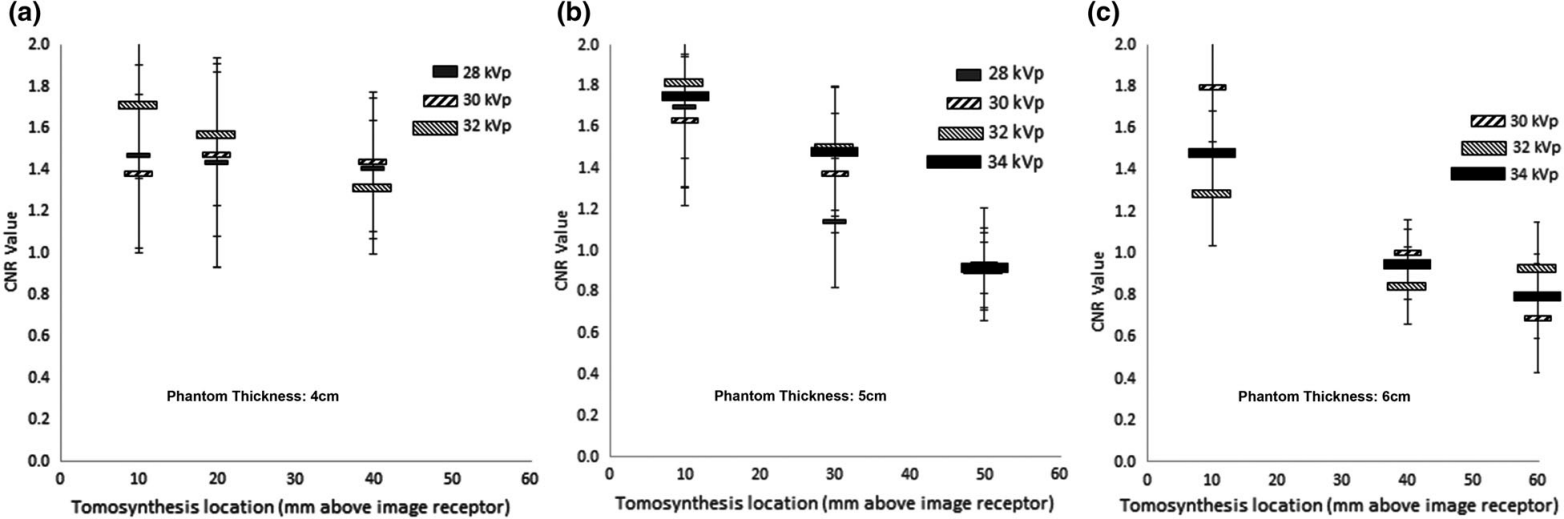
Contrast‐to‐noise ratio (CNR) values at heights above the detector. Error bars show the standard error of the mean (SEM). CNR values with a phantom thickness of (A) 4 cm; (B) 5 cm & (C) 6 cm.

**Figure 4:**
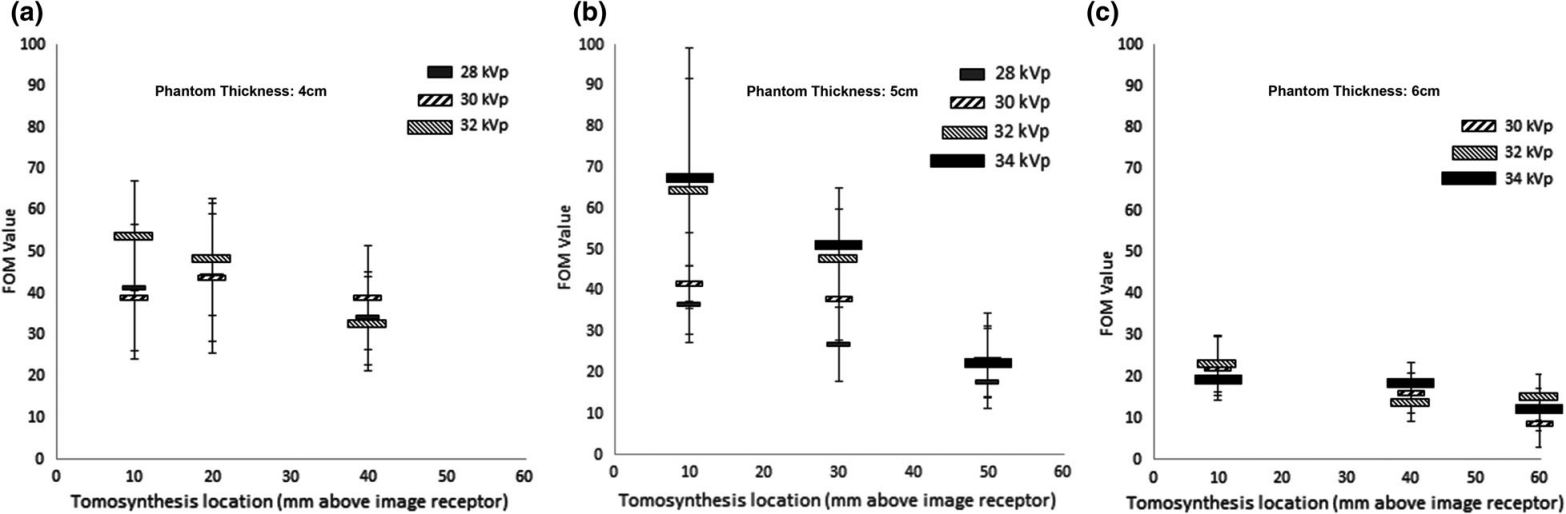
Figure‐of‐merit (FOM) values at heights above the detector. Error bars show the standard error of the mean (SEM). FOM values with a phantom thickness of a) 4 cm; b) 5 cm & c) 6 cm.

Measurements were obtained from the six spheroidal masses of each of the three tomosynthesis images. For each DBT image acquisition, a total of 18 spheroidal masses contributed to the CNR and FOM measurements for each selected tube voltage, phantom thickness and in each target slab height/tomosynthesis image slice height above the detector. Measurements of the CNR and FOM for all spheroidal masses within each image at each slab height and phantom thickness were made. Table 2 shows the mean and SEM of the CNR and FOM values from the spheroidal masses in the 30 DBT images. Figures 3A–C and 4A–C, respectively, show, in a graphical format, the CNR and FOM information provided in Table 2. Table 3 provides *P*‐value measures of the significance of the difference between the CNR and FOM measurements at various tomosynthesis image slice heights above the detector for 4, 5 and 6 cm phantom thicknesses combined for all tube voltage settings.

**Table 2:** Mean and standard error of the mean (SEM) values of the CNR and FOM measurements at various tomosynthesis target slab heights above the detector for 4, 5 and 6 cm phantom thicknesses and at selected tube voltage settings for each phantom thickness.

Phantom Thickness (cm)	Tube Voltage (kVp)	Average Glandular Dose (mGy) and (range)	Height above Detector (mm)	CNR		FOM	
				Mean	*SEM*	Mean	*SEM*
4	28	1.71 (1.69 to 1.76)	10	1.46	0.44	41.3	15.3
			20	1.43	0.50	44.2	18.6
			40	1.41	0.34	34.1	11.2
	30	1.46 (1.43 to 1.49)	10	1.38	0.38	38.9	14.8
			20	1.47	0.40	43.8	15.5
			40	1.44	0.34	38.8	12.5
	32	1.27 (1.25 to 1.29)	10	1.71	0.35	53.8	13.2
			20	1.57	0.34	48.2	13.5
			40	1.32	0.32	32.6	11.3
5	28	2.20 (2.10 to 2.25)	10	1.70	0.25	36.6	9.4
			30	1.14	0.31	26.8	9.1
			50	0.91	0.20	17.5	6.2
	30	1.80 (1.80 to 1.80)	10	1.64	0.32	41.7	12.4
			30	1.38	0.29	37.9	9.9
			50	0.93	0.27	22.9	11.6
	32	1.50 (1.50 to 1.50)	10	1.81	0.51	64.4	27.2
			30	1.50	0.30	47.8	12.0
			50	0.91	0.18	22.5	8.7
	34	1.35 (range 1.35 to 1.35)	10	1.75	0.54	67.4	31.7
			30	1.48	0.31	51.1	13.8
			50	0.92	0.12	22.4	8.4
6	30	2.20 (2.10 to 2.25)	10	1.80	0.30	22.0	7.6
			40	1.00	0.16	16.1	4.8
			60	0.69	0.26	8.6	5.6
	32	1.80 (1.80 to 1.80)	10	1.29	0.25	23.2	6.8
			40	0.84	0.18	13.9	4.6
			60	0.93	0.22	15.1	5.5
	34	1.50 (1.50 to 1.50)	10	1.48	0.20	19.2	3.8
			40	0.95	0.17	18.4	5.1
			60	0.80	0.20	12.1	5.0

**Table 3:** 2 × 2 contingency tables to compare CNR and FOM measurement differences between various target slab heights above the detector for 4, 5 and 6 cm phantom thicknesses. CNR and FOM measurements are the combined measurements at all tube voltage settings. The *P*‐values are shown, and significance differences between measurements at various slice heights are seen when *P* < 0.05.

Phantom Thickness		CNR: *P*‐value column vs. row		FOM: *P*‐value column vs. row	
4	Target Slab Location	20 mm	40 mm	20 mm	40 mm
	10 mm	0.995	0.894	0.998	0.663
	20 mm	‐	0.931	‐	0.662
5	Target Slab Location	30 mm	40 mm	30 mm	40 mm
	10 mm	0.235	0.001	0.513	0.011
	30 mm	‐	0.091	‐	0.157
6	Target Slab Location	40 mm	60 mm	40 mm	60 mm
	10 mm	0.004	0.000	0.433	0.079
	40 mm	‐	0.750	‐	0.601

## Discussion

One measure of image quality for images is the CNR measurements.^34^, ^35^, ^36^, ^38^ For each measure of the CNR from each of the 30 image acquisitions, three tomosynthesis images were selected, and a total of 18 objects contributed to the measurements. Approximately, 2500 pixel values contributed to the object measurement and 3700 pixel values to the background measurement.

When the CIRS breast phantom consisted of four slabs, each having a thickness of 40 mm, there was no significant difference in the CNR and FOM measurements (see Table 3 for *P*‐values) when the DBT slices were obtained near the detector, near the middle of the phantom or near the top of the phantom. However, when the CIRS breast phantom was 5 cm thick, there was a significant reduction (*P* = 0.001) of image CNR values obtained the near top of the phantom to those obtained near the bottom of the phantom. There was no significant difference (*P* = 0.235) between CNR values obtained near the bottom and middle of the phantom. Furthermore, when the phantom was 6 cm thick, there were significant reductions of CNR values obtained near the middle and top (*P* = 0.001 and *P* = 0.000), compared to those obtained near the bottom of the phantom.

Post hoc analysis shows that the differences in CNR values that have been demonstrated between differing CNR values obtained from images at differing heights above the detectors can not be attributed to the differing tube voltage. The differences in CNR values can be attributed to the phantom thickness and the height of the tomosynthesis slice above the detector.

These findings are consistent with those reported by Cockmartin et al.^27^ In their work, they reported that DBT was worse at detecting microcalcifications when breast thicknesses were greater than 40 mm and DBT slice locations were 20 mm or more above the height of the detector.

Measurements of FOM are used to evaluate image quality; in this case, CNR measurements, against the dose used to obtain the image.^20^, ^37^, ^38^ In digital radiographic imaging, including digital mammography, it is well‐accepted that as dose increases in the image acquisition, image quality also increases.^39^, ^40^ For all DBT image acquisitions, that is at each tube voltage setting and phantom thickness, the automatic exposure control system determined the tube current‐exposure time product and hence the dose that was delivered. With one exception, there was no significant difference (*P* > 0.05) between FOM values obtained from the tomosynthesis slice image at differing heights above the detector. The exception was there was a difference (*P* = 0.011) at a phantom thickness of 5 cm and at heights above the detector between 10 and 50 mm. It can be generally concluded that the dose delivered during the image acquisition is not playing a role in determining the difference in image quality at various tomosynthesis slice image heights above the detector. A limitation of this study is that the dose measures used in the calculation of FOM was AGD. The AGD is a measure for all images in each DBT image set, and as such the ADG for each image slice does not differ when comparing images in the same DBT image set.

The purpose of this preliminary study was to determine if anecdotal comments about image quality differences at different heights in DBT were correct. The measures of image quality used, that of the CNR and FOM, are not necessarily related to clinical performance. The study by Cockmartin et al^27^ used observers to score the presence or absence of the spheres and microcalcifications. Their approach replicates clinical conditions. The study by Cockmartin et al found that when breast thicknesses were 40 mm and greater and when the DBT image slice was 20 mm or greater above the height of the detector, microcalcification detection became worse in DBT than in DM.

Possible causes of the results reported in this study are many. These could include the reconstruction algorithms not being optimised at greater heights above the detector; differences due to physical angular variations at greater heights above the detector; the effective focal spot size variations due positional changes as the X‐ray tube moves in an arc or the effects of increased scatter radiation as the phantom thickness increases. The methodology chosen for this study was designed to review results of a single measure of image quality. Now that these results are known, they need to be confirmed by other measures of image quality, such as the use of observers, and other studies need to be undertaken to determine the cause of these results.

Further limitations of this study are that only one mammography unit was used to acquire the DBT images. Furthermore, only one measure of image quality, that of the CNR, was used to evaluate the DBT images at differing heights above the detector. An additional limitation is that only one phantom diameter size was used. As breast thickness increases, typically breast diameter and SPR also increase.

## Conclusion

In DBT, image quality as measured by the CNR varies between tomosynthesis slice image heights above the detector when phantom thickness is greater than 4 cm thick. Image quality of the DBT images towards the top of the phantom was less than images obtained nearer to the detector. The variations in the CNR that occurred were due to increased phantom thickness and not due to tube voltage setting or the delivered dose. FOM, with the method used in this study, did not generally show any differences in measurements that resulted from variations between tomosynthesis slice image heights above the detector and between phantoms of different thicknesses.

Clinicians need to be aware of this preliminary finding as a potential source of false‐negative findings when using DBT. In breast screening programs which use DBT, this finding could also affect recall rates.

As the CNR is a single measure of image quality and is not necessarily related to clinical performance, further research in DBT image quality when the images are obtained at differing heights above the detector, including observer studies, is needed. It is recommended that the focus of those studies needs to be when breast or phantom size is greater than 4 cm thick.

## Conflict of Interest

The authors declare that they have no conflicts of interest.
